# Presenting ten thousand research articles

**DOI:** 10.1038/ncomms12816

**Published:** 2016-08-18

## Abstract

*Nature Communications* celebrates its 10,000th published article and introduces changes to improve our service to authors and readers

A few weeks ago we celebrated a milestone at *Nature Communications*, our 10,000th published research article—an incredible achievement for a journal that is just over six years old. Together, these thousands of papers have received more than 97 million page views and have been quoted in over 22,000 news pieces according to Altmetrics. Needless to say, we are extremely grateful to our authors, for publishing some of their best work in our journal, and to our reviewers, without whom this success would not have been possible.

Along with the rapid growth, the journal has changed considerably over these years. We launched the journal with four editors, and at that time each of them had to oversee a wide range of subject areas given the breadth of our scope. Now, our editorial team is made up of more than 60 editors based in three offices across the world—in London, Shanghai and New York. The breadth of expertise and nationalities among our team is a vivid reflection of the diversity of content in our pages. To accommodate and thrive in this dynamic environment, we have adapted our internal structure and workflows to better cater to the needs of our authors. In our editorial department, we organized ourselves into small thematic teams of editors, led by team managers, that cover areas such as neuroscience, cancer research, fundamental physics, Earth sciences, organic chemistry and more. These editorial teams represent centres of expertise and ensure a consistent and expert handling of manuscripts across disciplines. As a result of the expansion of the editorial team, we have taken the decision to disband our Editorial Advisory Panel, who were a huge support in the early years of the journal. We would like to take this opportunity to thank all the scientists who contributed their time and expertise during their tenure on our panel.

Looking outward, we have now begun to adapt our web site to a more modern look. Initially designed for a journal with a small number of published papers and created at a time before smart tablet devices came on the market, our online presence had failed to catch up with the changing demands of our authors and readers. In the next few weeks, we will be moving the majority of our users to a new design, which is optimized for different device platforms and hopefully will improve the discoverability of our content.

**We aim to provide an excellent service to our authors, tailored to the needs of each of the research areas that we cover.**

The benefit of an online-only journal is that we can publish any paper fulfilling our editorial criteria, irrespective of other limiting factors such as page budgets, but this raises important problems of content discovery. Ours is a large journal, which published 3,193 articles in 2015 alone, and it is of utmost importance for us that each and every published paper receives the online attention that it deserves, and can be accessed and discovered by as many readers as possible. To improve the discoverability of our content, we now provide a more structured way to browse our papers by subject area at http://www.nature.com/ncomms/browse-subjects. Furthermore, our output now also appears on *Nature*'s subject category pages at http://www.nature.com/subjects, along with that of our sister journals. These pages allow cross-journal subject browsing and provide readers with the option of subscribing to subject-specific RSS feeds of cross-journal output.

In addition, we are also aiming to highlight some of our best content on our web site. An example is the featured image, which now stretches across the top part of the web page. Going forward, we welcome suitable suggestions from authors of our newly accepted papers to feature on this prominent slot of our home page.

We hope you like our new face online and these initiatives. In particular, we welcome feedback so that we can continue to improve our service to you—reader, reviewer and author—and to increase the impact our papers make to science and society at large.

Published online: 18 Aug 2016

